# Chronic Niche Inflammation in Endometriosis-Associated Infertility: Current Understanding and Future Therapeutic Strategies

**DOI:** 10.3390/ijms19082385

**Published:** 2018-08-13

**Authors:** Yi-Heng Lin, Ya-Hsin Chen, Heng-Yu Chang, Heng-Kien Au, Chii-Ruey Tzeng, Yen-Hua Huang

**Affiliations:** 1 Department of Biochemistry and Molecular Cell Biology, School of Medicine, College of Medicine, Taipei Medical University, Taipei 11031, Taiwan; b101099158@tmu.edu.tw (Y.-H.L.); kg230811100100@gmail.com (Y.-H.C.); hychang@tmu.edu.tw (H.-Y.C.); 2 TMU Research Center for Cell Therapy and Regeneration Medicine, Taipei Medical University, Taipei 11031, Taiwan; 3 Graduate Institute of Medical Sciences, College of Medicine, Taipei Medical University, Taipei 11031, Taiwan; 4 International PhD Program for Cell Therapy and Regeneration Medicine, College of Medicine, Taipei Medical University, Taipei 11031, Taiwan; hk.au@msa.hinet.net; 5 Department of Obstetrics and Gynecology, School of Medicine, College of Medicine, Taipei Medical University, Taipei 11031, Taiwan; tzengcr@tmu.edu.tw; 6 Center for Reproductive Medicine, Taipei Medical University Hospital, Taipei Medical University, Taipei 11031, Taiwan; 7 Department of Obstetrics and Gynecology, Taipei Medical University Hospital, Taipei 11031, Taiwan; 8 Comprehensive Cancer Center of Taipei Medical University, Taipei 11031, Taiwan; 9 The PhD Program for Translational Medicine, College of Medical Science and Technology, Taipei Medical University, Taipei 11031, Taiwan

**Keywords:** endometriosis, infertility, niche, inflammation, immunomodulation, mesenchymal stem cell

## Abstract

Endometriosis is an estrogen-dependent inflammatory disease that affects up to 10% of women of reproductive age and accounts for up to 50% of female infertility cases. It has been highly associated with poorer outcomes of assisted reproductive technology (ART), including decreased oocyte retrieval, lower implantation, and pregnancy rates. A better understanding of the pathogenesis of endometriosis-associated infertility is crucial for improving infertility treatment outcomes. Current theories regarding how endometriosis reduces fertility include anatomical distortion, ovulatory dysfunction, and niche inflammation-associated peritoneal or implantation defects. This review will survey the latest evidence on the role of inflammatory niche in the peritoneal cavity, ovaries, and uterus of endometriosis patients. Nonhormone treatment strategies that target these inflammation processes are also included. Furthermore, mesenchymal stem cell-based therapies are highlighted for potential endometriosis treatment because of their immunomodulatory effects and tropism toward inflamed lesion foci. Potential applications of stem cell therapy in treatment of endometriosis-associated infertility in particular for safety and efficacy are discussed.

## 1. Introduction

Endometriosis is an estrogen-dependent inflammatory disease characterized by the presence of endometrial glands and stroma outside the uterine cavity. It affects 5–10% of women of reproductive age, up to 80% of women with pelvic pain, and 20–50% of women with infertility [1,2]. Affected women experience impaired quality of life due to chronic pelvic pain and other clinical symptoms such as dysmenorrhea, menorrhagia, dyspareunia, dysuria, and dyschezia [3]. Endometriosis is also associated with increased risk of certain cancer types and other chronic diseases, including ovarian and endometrial cancer [4,5], cardiovascular diseases [6], autoimmune diseases [7], and allergic disorders [8].

Despite its prevalence and correlation with several diseases, the exact pathogenic mechanism of endometriosis remains unclear. Development of endometriosis may be the endpoint of several combined aberrant biological processes. The most plausible hypothesis is retrograde menstruation, where endometrial fragments regurgitated through the fallopian tubes during menstruation are subsequently implanted in secondary sites [9]. Other possible cellular and molecular mechanisms include coelomic metaplasia, lymphovascular spread, endometrial stem cell implantation, and immune dysregulation [9,10]. All of these theories complementarily explain the complicated and variable nature of endometriosis development and progression.

Current treatment for endometriosis focuses on infertility and pain management. For patients with suspected endometriosis based on presented symptoms and signs, many clinicians begin empirical treatment before making a definitive diagnosis, using medical therapies such as nonsteroidal anti-inflammatory drugs, hormonal contraceptives, progestogens, antiprogestogens, gonadotropin-releasing hormone (GnRH) agonists and antagonists, and aromatase inhibitors [11,12]. These reagents function by inducing hypoestrogenism, amenorrhea, or endometrial atrophy [13]. When empirical therapies fail to alleviate symptoms or long-term medical treatment is warranted, laparoscopic exploration, excision, and adhesiolysis may be performed for definitive diagnosis and curative treatment [14].

Medical management effectively reduces pain in most endometriosis patients. However, for infertility treatment, hormonal medical therapies alone are inadequate. Because these therapies suppress ovarian function and create a contraceptive state along with endometrial atrophy, they do not benefit patients seeking pregnancy. Hughes et al. showed that ovulatory suppressive medications such as oral contraceptive pills, GnRH agonists, and danazol did not improve spontaneous pregnancy and live birth rates for infertile women with endometriosis seeking conception [15]. Currently, conventional medical therapy plays a role only in treating endometriosis-associated infertility in assisted reproductive technology (ART); it was demonstrated that pretreatment with GnRH agonist for 3–6 months before initiation of in vitro fertilization (IVF) or intracytoplasmic sperm injection could improve the pregnancy rate 4-fold [16]. It has been suggested that long-term use of GnRH agonists could improve endometrial receptivity by reducing aromatase and cyclooxygenase (COX)-2 expression in a eutopic endometrium [17]. Using cryopreserved embryo transfer instead of fresh embryos further improves IVF outcomes by circumventing the excessive ovarian suppression caused by long-term GnRH agonist treatment [18,19]. The aromatase inhibitor letrozole may also be used to improve IVF outcomes in patients with low expression of endometrial integrin αvβ3; this is a common finding in endometriosis cases [20]. Novel nonhormonal medical agents that target other pathways such as inflammation and angiogenesis to treat endometriosis-associated infertility are currently under investigation.

Although the cause of endometriosis-induced infertility remains elusive, several causes have been proposed to explain it, including distorted pelvic anatomy due to adhesions, ovarian dysfunction, defective peritoneal function, and altered endometrial receptivity [21]. Immune dysfunction plays a role in each of these causes. In this review, we first examine the dysregulated niche immune modulation in each anatomical compartment, and then discuss novel treatment strategies that target immune pathways to restore fertility in endometriosis patients.

## 2. Chronic Niche Inflammation in Endometriosis Development

**Figure 1:**
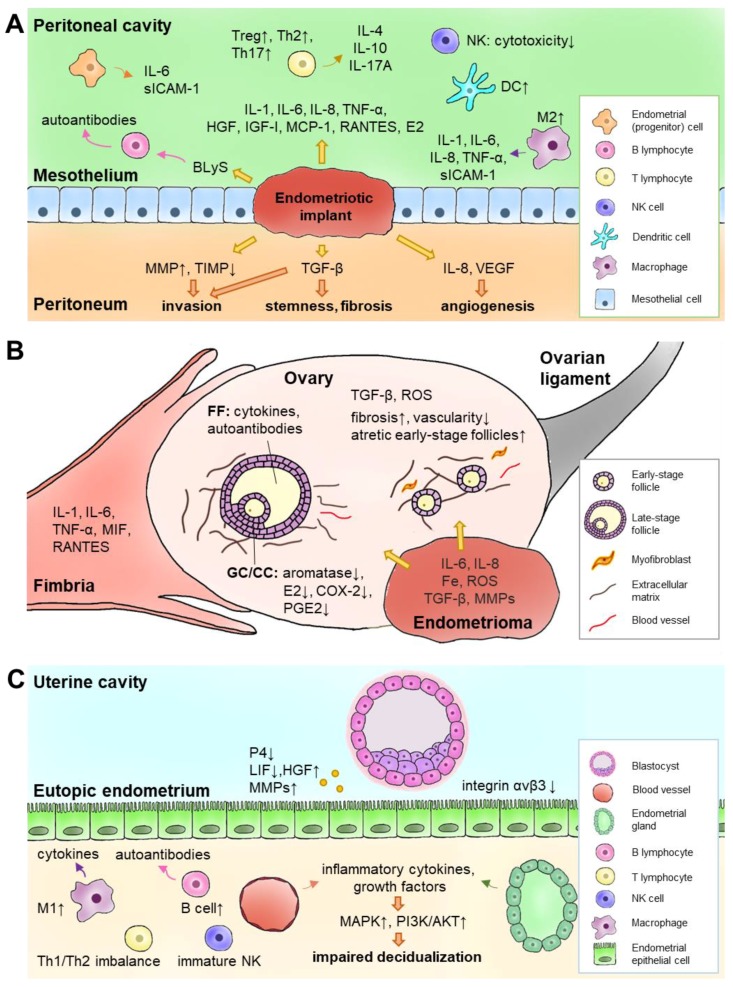
Different inflammatory niche in (**A**) peritoneal cavity, (**B**) ovary, and (**C**) eutopic endometrium in endometriosis. The population of each immune cell type, the level of cytokine/hormone/protein expression, and the activation of cellular pathways are depicted by an up arrow or a down arrow to represent an increase or a decrease, respectively. BLyS, B lymphocyte stimulator; CC, cumulus cell; COX-2, cyclooxygenase 2; DC, dendritic cell; E2, estradiol; FF, follicular fluid; GC, granulosa cell; HGF, hepatocyte growth factor; IGF-I, insulin-like growth factor 1; IL, interleukin; LIF, leukemia inhibitory factor; MAPK, mitogen-activated protein kinase; MCP-1, monocyte chemoattractant protein-1; MIF, macrophage migration inhibitory factor; MMP, matrix metalloproteinases; NK, natural killer; P4, progesterone; PGE2, prostaglandin E2; PI3K, phosphoinositide 3-kinase; RANTES (CCL5), regulated on activation, normal T cell expressed and secreted; ROS, reactive oxygen species; sICAM-1, soluble intercellular adhesion molecule-1; TNF-α, tumor necrosis factor alpha; TGF-β, transforming growth factor beta; Th, T helper cell; Treg, regulatory T cell; VEGF, vascular endothelial growth factor.

The tissue niche provides several chronic inflammatory environments for endometriosis development, particularly in the peritoneal cavity, ovaries, and uterus (Figure 1).

### 2.1. Peritoneal Cavity

The peritoneal cavity is immersed in peritoneal fluid, which is mainly ovarian exudate produced by developing follicles and corpus luteum [22]. Its volume and content vary significantly in different phases of the menstrual cycle because vascular permeability increases with estrogen concentration. Peritoneal fluid contains electrolyte, urea, ovarian steroidal hormones such as estrogen and progesterone, and cellular components including endometrial cells, macrophages, lymphocytes, and red blood cells. These cellular components may have their own secretions; for example, endometrial cells secrete glycodelins, and macrophages can produce cytokines and growth or angiogenic factors [22].

Impaired fertility in endometriosis patients mostly results from a chronic inflammatory state caused by an abnormal peritoneal environment. The volume of peritoneal fluid is significantly higher in infertile women with endometriosis than in those without the disease [23]. Numerous aspects of the immune system are altered in endometriosis, including inhibited T-cell-mediated cytotoxicity to endometrial cells, low natural killer (NK) cell activity, and increased numbers of activated macrophages and proinflammatory cytokines [24]. These changes create an oxidative and immunotolerant microenvironment for endometriotic implants to survive [25,26].

The interaction between an endometriotic lesion and its local immune environment was reviewed [1]. Endometriotic implants secrete estradiol, progesterone, monocyte chemoattractant protein (MCP)-1, transforming growth factor (TGF)-β, vascular endothelial growth factor (VEGF), and proinflammatory cytokines such as interleukin (IL)-1, IL-6, and IL-8, and tumor necrosis factor alpha (TNF-α), among others [1]. This cocktail of secretions in peritoneal fluid promotes a proliferative and angiogenic environment that enhances endometriosis development and progression. Endometrial cells exposed to the peritoneal fluid of endometriosis patients have been shown to upregulate their VEGF and plasminogen activator gene expression [27,28]. Because fertilization occurs in the ampulla of the fallopian tube with exposure to peritoneal fluid, changes in peritoneal fluid composition directly influence the fertilization process. For example, endometriosis may impair sperm mobilization through macrophage-secreted IL-1, IL-6, and macrophage migration inhibitory factor (MIF) [29,30]. Moreover, TNF-α can damage sperm DNA by inducing apoptosis and oxidative stress [31], and sperm–oocyte binding and fusion are disturbed by TNF-α, IL-1, MIF, and regulated on activation, normal T cell expressed and secreted (RANTES)/C-C motif chemokine ligand 5 (CCL5) [30,32].

Cross-talk between the immune and endocrine systems has been observed in endometriosis because endometriotic lesions are characterized by excessive local estrogen production, abnormal estrogen receptor (ER) expression, and increased cyclooxygenase 2 (COX-2) and prostaglandin expression. Zhao et al. demonstrated that targeting either ER-α or ER-β with isoform-specific ligands significantly reduced inflammation markers such as IL-6, TNF-α, nuclear factor kappa B (NFκB), COX-2, C-C motif chemokine ligand 2 (CCL2), and RANTES and decreased T-cell and macrophage infiltration in endometriotic implants [33]. Burns et al. showed that the development and maintenance of endometriosis could be divided into an early immune-predominant initiation phase mediated by IL-6 signaling and an ensuing hormone-predominant phase mediated by estrogen signaling [34].

Endometriosis is characterized by enhanced humoral immune response with increased B lymphocyte numbers and autoantibody production [35]. Immunohistochemical and gene expression microarray analysis revealed that endometriotic lesions were abundant in plasma cells and activated macrophages, with highly expressed cytokine B lymphocyte stimulator (BLyS) [36]. BLyS is a ligand of the TNF cytokine family that plays a major role in B lymphocyte differentiation, homeostasis, and maturation, and its overexpression is associated with autoimmune diseases [37]. High levels of BLyS result in B lymphocyte production of a plethora of autoantibodies. These autoantibodies include antiendometrial, anti-DNA, antiphospholipid, and antinuclear antibodies [38], and two of them have been identified as antiendometrial autoantibodies against alpha 2-HS glycoprotein and transferrin [39]. Because transferrin functions as an iron transporter in the human body, a high transferrin level in peritoneal fluid could reflect a high iron level in peritoneal fluid in endometriosis patients [39]. The presence of excessive iron in peritoneal fluid could induce oxidative stress and cause tissue injury and subsequent de novo lesion formation; moreover, an autoimmune reaction against transferrin interferes with iron removal from the peritoneal cavity and affects granulosa cell maturation in the ovaries [40]. The presence of autoantibodies also reduces sperm motility [41].

Identifying biomarkers that can assist in noninvasive diagnosis and monitoring of endometriosis remains a challenge because of the heterogeneous nature of the disease process and presentation and the interference of comorbidities, and there has been insufficient evidence to recommend any biomarker for routine clinical practice [42,43]. Numerous studies have analyzed the peritoneal fluid of endometriosis patients to identify cytokine signatures characteristic of the disease. Rakhila et al. demonstrated a distinct cytokine expression profile favoring proliferation and angiogenesis for patients with endometriosis who showed increased levels of EGF, FGF-2, IL-1α, MIP-1β, transforming growth factor (TGF)-α, platelet-derived growth factor (PDGF)-AA, PDGF-BB, monocyte chemotactic protein 3 (MCP-3), soluble CD40 ligand (sCD40L), C-X-C motif chemokine ligand 1/2/3 (Gro Pan), IL-17α, macrophage-derived chemokine (MDC), and RANTES [27]. Several studies also indicated an increased IGF system in the peritoneal environment of endometriosis patients, leading to enhanced endometrial stromal cell proliferation [44,45,46,47]. 

Beste et al. similarly demonstrated that an endometriosis subset had a distinct cytokine profile with IL-1ra, IL-1β, IL-6, IL-8, IL-10, IL-16, hepatocyte growth factor (HGF), MCP-1, MIF, monokine induced by gamma interferon (MIG), granulocyte-colony stimulating factor (G-CSF), growth regulated oncogene (GRO)-α, and RANTES [48]. Network analysis revealed that this cytokine signature was associated with a macrophage-driven process related to NFκB, Jun proto-oncogene (c-Jun), Fos proto-oncogene (c-Fos), activator protein 1 (AP-1), and mitogen-associated kinase signaling [48]. Another study by the same authors aimed to identify endometriosis patients among women assessed for infertility, and concluded that a panel of 11 cytokines (IL-5, IL-9, IL-13, IFN-α2, cutaneous T-cell attracting chemokine (CTACK), HGF, MCP-1, MCP-3, M-CSF, leukemia inhibitory factor (LIF), and SCGF-β) was distinguishable in infertile patients with endometriosis; this profile suggested that dysregulated Th1/Th2 activity could underlie endometriosis-associated infertility [49]. However, whether these aberrant cytokine expressions are the cause or a consequence of the disease remains to be explored, and more studies are warranted to better stratify patients and assess the applicability of these cytokine profiles in clinical use.

### 2.2. Ovaries

Ovarian endometriomas could alter ovarian function through space-occupying and local effects [50]. The cystic fluid within an endometrioma is a rich source of proinflammatory cytokines (IL-6, IL-8), iron, reactive oxygen species (ROS), growth factors such as TGF-β, and matrix metalloproteases (MMPs) [50]. These cystic fluid contents could affect adjacent ovarian function through diffusion into surrounding tissue. Structural alterations were identified in the neighboring tissue of an ovarian endometrioma, with lower follicular density, greater fibrosis, and loss of cortex-specific stroma [51]. In addition, cortical biopsies of ovaries containing endometriomas revealed atresia of early follicles with increased cleaved caspase-3 immunostaining, which was not seen on contralateral ovaries without endometriomas [52]. Increased oxidative stress due to local inflammatory reactions has been observed in the ovarian cortex surrounding endometriomas [52,53]. Both TGF-β1 and ROS promote fibrosis and adhesion formation through myofibroblast differentiation and profibrotic gene expression of plasminogen activator inhibitor-1 [54]. Structural change including loss of ovarian stroma has a detrimental effect on folliculogenesis due to reduced blood supply to follicles and decreased growth factors secreted by stromal cells [55].

Follicular fluid forms a distinct microenvironment within the ovaries and contains cytokines produced by granulosa and local immune cells. Most studies on the follicular fluid of endometriosis patients have been recruited with patients undergoing IVF following stimulation of gonadotropin. Such reports have suggested high follicular levels of IL-1β, IL-6, IL-8, and IL-18. One study determined that the follicular fluid of naturally matured follicles (without gonadotropin stimulation) in endometriosis patients had higher IL-1β and IL-6. The abnormal presence of intrafollicular IL-1β and IL-6 could affect the follicular biology of endometriosis patients because low intrafollicular IL-6 is associated with a higher pregnancy rate [56]. Proinflammatory cytokines can have detrimental and beneficial effects, and high intrafollicular IL-1β has been found to correlate with higher fertilization and implantation rates [57]. High levels of IL-8, IL-12, and adrenomedullin were detected in one study, correlating negatively with oocyte maturity and embryo quality [58]. Although IL-18 was highly expressed in the peritoneal fluid of endometriosis patients, high amounts were not observed in the follicular fluid of such patients [59]. Inflammatory cytokines could affect folliculogenesis in endometriosis patients by regulating G protein-coupled estrogen receptor (GPER) expression and the fraction of GPER positive macrophages in the ovaries [60]. GPER is a transmembrane receptor that participates in nongenomic estrogen signaling pathway and is upregulated in endometriotic lesion and eutopic endometrium of endometriosis patients [60,61]. GPER signaling plays an important role in follicular maturation, and the lower follicular expression of GPER could explain the lower follicular count in endometrioma patients [60,62]. In moderate/severe endometriosis patients, the follicular environment was also characterized by increased oxidative stress and leukocyte activation marker myeloperoxidase, and this increase was correlated with decreased oocyte quality and fertility [63]. Hormone status in the follicular microenvironment is less certain, with several studies producing different results [59,64].

Both ovarian reserve and response to controlled ovarian stimulation are compromised in endometriosis patients, especially in more severe cases (American Society for Reproductive Medicine, ASRM stage III/IV) [65,66]. In a meta-analysis examining 1039 patients, those with endometriosis had decreased oocyte retrieval, lower metaphase II oocyte numbers, and fewer embryos formed compared with the control group [67]. However, the number of oocytes retrieved from the diseased side of the ovary, the number of metaphase II oocytes, and the embryo count were all similar to those of the healthy contralateral side of the ovary [67]. Significant differences from the healthy contralateral ovary appeared when the endometrioma was large (≥5 cm) [68]. Whether poorer ovarian reserve and response are due to endometriosis or surgical injury to the ovaries during cystectomy are still under debate [69]. Furthermore, coexisting deep-infiltrating endometriosis was shown to further deteriorate ovarian reserve and oocyte retrieval compared with endometrioma alone [70].

### 2.3. Uterus

Whether endometriosis affects the eutopic endometrial lining to influence implantation has been much debated [71,72]. Several clinical studies have indicated a decreased implantation rate in endometriosis patients undergoing IVF [73,74,75], and a meta-analysis of 27 studies demonstrated significantly lower implantation and clinical pregnancy rates in patients with severe endometriosis [76]. To exclude factors associated with oocyte and embryo quality, a prospective study using sibling oocytes from the same donor demonstrated that recipients with endometriosis had significantly lower implantation and pregnancy rates than control recipients [77]. However, not all related studies have reached the same conclusion, instead generally attributing poorer IVF outcome in endometriosis patients to compromised oocyte and embryo quality [78,79,80,81]. In a large retrospective cohort study involving 22,416 women with or without endometriosis undergoing IVF, no significant differences between groups was found for live birth, clinical pregnancy, and miscarriage rates [81]. Women with endometriosis had significantly lower retrieved oocyte numbers, even though the number of fertilized oocytes did not differ after adjustment for retrieved oocyte numbers [81]. An age-stratified analysis found no difference in reproductive outcomes for women in the under-35 and 35–40 age groups [81]. Thus, the effect of endometriosis on implantation remains debatable. Possible explanations for discrepancies among studies include different research designs, highly heterogeneous inclusion criteria, and low numbers of enrolled patients.

The effect of endometriosis on endometrial receptivity is based on inflammation. Progesterone exhibits anti-inflammatory activity, and in normal circumstances, an inflammatory response occurs after progesterone withdrawal in the late secretory phase of the menstrual cycle. Decreased progesterone leads to decreased prostaglandin metabolism and increased ROS, which activate an NFκB-mediated inflammatory cascade that induces the processes required for menstruation [82]. The progesterone resistance that characterizes endometriosis mimics this late secretory phase, leading to premature initiation of inflammation [71]. The result is accumulation of proinflammatory cytokines, chemokines, ROS, MMPs, COX-2, and prostaglandins in the endometrium, with all of these inflammatory mediators feeding on one another in a positive feedback loop. Immune transcriptomic profiling showed that compared with a healthy control endometrium, the eutopic endometrium in endometriosis patients was dysregulated for genes related to cytokine and chemokine production, cell adhesion, apoptosis regulation, and wound-healing response [83]. In addition, the eutopic endometrium was also downregulated for genes related to decidualization such as NOTCH1 and NOTCH2 compared with a control endometrium [83]. Dysregulated gene expression related to immune activity has been observed, including increased B-cell signaling and a decreased Treg population [83,84]. To determine the dominant T-cell subtype in infertile endometriosis patients, Koval et al. studied the expression of transcription factors related to differentiation of various subtypes in eutopic endometrial tissues and found increased expression of T-box expressed in T cells (T-bet) and GATA-binding protein 3 (GATA3), the transcription factors involved in Th1 and Th2 differentiation, and decreased expression of forkhead box P3 (Foxp3), which drives Treg differentiation [85]. Abundant immature uterine natural killer cells in the eutopic endometrium also characterized endometriosis patients with infertility [86].

Although the endometrium of a woman with endometriosis is morphologically identical to that of a woman without this disease, it exhibits altered biochemical responses during implantation. Progesterone resistance is generally observed in endometriosis, with altered progesterone receptor (PR) composition for decreased PR-β isoform expression [87]. Abnormally elevated ER-α isoform also occurs in endometriosis patients in the mid-secretory stage, leading to implantation failure [88]. The eutopic endometrium of a woman with endometriosis contains low, but significant, levels of P450 aromatase enzyme activity, which enhances local estrogen activity [89]. Stromal cells derived from the endometrium of a woman with endometriosis have a reduced decidualization capacity [90], possibly because of progesterone resistance and inflammatory cytokines TNF-α and IL-1 [91]. Several preclinical studies have shown that the inflammatory microenvironment maintained by endometriosis compromises implantation ability in animal models. Intraperitoneal injection of peritoneal fluid collected from infertile women with endometriosis decreased the implantation rate in a rodent model, as well as endometrial integrin αvβ3 and LIF expression [92]. During a normal implantation process, endometrial epithelial expression of homeobox A10 (HOXA10) and HOXA11 increases in the luteal phase, but this fails to occur in endometriosis because of dysregulated promoter methylation [93]. Endometrial receptivity is related to integrin expression of the endometrium; however, women with endometriosis have reduced expression of αvβ3, possibly due to decreased HOXA10 [93]. Other implantation-related biomarkers such as glycodelin A, osteopontin, LIF, lysophosphatidic acid receptor 3, and insulin-like growth factor binding protein 1 (IGFBP1) are also affected [94]. Expression of empty spiracles homolog 2 (EMX2) was elevated during the implantation window in endometriosis patients and associated with implantation failure [95]. All of these abnormal gene expressions alter the endometrial receptivity of endometriosis patients. However, none of these individual endometrial markers have been successfully translated to clinical use for receptivity prediction.

Endometrial receptivity array (ERA) has emerged as a new diagnostic tool for determining a woman’s implantation window by simultaneously examining the transcriptional expression of a panel of 238 genes related to endometrial receptivity [96]. ERA can guide clinicians to the optimal time frame for performing embryo transfer in recurrent implantation failure patients, and has been demonstrated to outperform endometrial histologic examination [97,98]. Garcia-Velasco et al. conducted a pilot study comparing ERA results between endometriosis patients and healthy women; their results indicated no significant differences in any of the 238 genes in women with endometriosis, irrespective of the disease stage [99]. Therefore, the researchers concluded that endometriosis did not affect endometrial receptivity, at least on a transcriptomic level. Future integrated studies that can capture endometrial status at the proteomic, epigenomic, and hormonal levels may offer greater insight into the effect of endometriosis on the implantation process.

## 3. Targeting Inflammation for Treatment of Endometriosis-Associated Infertility

Current medical management of endometriosis only treats symptoms rather than cures the disease, and symptoms recur when medication is discontinued. To achieve more effective therapy, novel treatment strategies that target specific pathogenic mechanisms must be investigated. Because endometriosis is considered a chronic inflammatory disease, many studies have explored immunomodulatory agents for restoring balanced immune status. Although this review focuses on systemic pharmacologic treatments, tubal flushing with oil-based contrast media has also been shown to increase short-term pregnancy rate in endometriosis and infertile patients, possibly by increasing the uterine NK cell population and restoring endometrial osteopontin expression [100,101]. Table 1 summarizes drugs targeting inflammatory pathways that have been tested in clinical trials. 

### 3.1. Immunomodulators

Pentoxifylline treatment was found to reduce the endometriotic lesion size in an animal model [115]. In addition, periovulatory treatment with pentoxifylline was found to improve the fertilization rate in a rodent model of endometriosis [116]. However, there is still insufficient evidence supporting use of pentoxifylline in management of endometriosis patients with infertility [117]. Luflunomide also appeared to reduce endometriotic implant size in a rodent model [118]. Loxoribine, also an immunomodulator, was found to decrease endometriotic implant size in a rodent model with an increased number of dendritic cells and a decreased number of natural killer cells [119]. Rosiglitazone and pioglitazone are two drugs that belong to the class thiazolidinedione and are clinically used as insulin sensitizer for diabetes treatment [111]. They act as a peroxisome proliferator-activated receptor gamma (PPAR-γ) agonist that can modulate immune cell activity and cytokine secretion by affecting NFκB activity and have been shown to limit endometriotic lesion development in preclinical studies [120]. Kim et al. showed that pioglitazone could suppress RANTES secretion and improve embryo implantation in stage III and IV endometriosis patients receiving IVF therapy [111]. However, both rosiglitazone and pioglitazone have an adverse effect of developing myocardial infarction, and their use should be carefully assessed in patients predisposed to cardiovascular diseases [121]. Metformin is an antidiabetic drug that potentially can be repurposed for cancer treatment due to its mechanistic target of rapamycin (mTOR) inhibition, cytotoxicity, and immunomodulation effects [122]. Metformin’s immunomodulation effect is based on its ability to induce class switching of cytotoxic T cells [122]. Metformin has been found to reduce cytokine effect, aromatase activity, and StAR expression in preclinical endometriosis models, thus regulating local estrogen production [123,124]. In a clinical study involving 69 infertile stage I and II endometriosis patients, metformin was demonstrated to decrease serum cytokine levels and improve pregnancy rate [112]. 

Natural compounds such as resveratrol have also been studied for endometriosis treatment. Resveratrol is a polyphenolic compound isolated from plants and has been demonstrated to reduce inflammation and oxidative stress in preclinical endometriosis models [125,126]. Epigallocatechin gallate (EGCg) is a green tea extract that exerts immunomodulation activities via increased peritoneal phagocytic activity and NK cell cytolysis [127]. EGCg has been shown to inhibit angiogenesis, fibrosis, and endometriotic cell growth in preclinical models, and a phase II clinical trial is currently ongoing (Clinical Trials.gov ID: NCT02832271) [128].

### 3.2. Anticytokine Treatment

The peritoneal fluid of endometriosis patients is abundant in proinflammatory cytokines, particularly TGF-β, IL-6, and TNF-α [1]. Thus, targeting proinflammatory cytokines has been proposed as a treatment strategy. An initial study showed that inhibiting TNF-α-mediated pathways could reduce inflammatory cytokine production, metalloprotease expression, and epithelial–mesenchymal transition markers of endometriotic cells [129]. Results from clinical studies have not supported using anti-TNF-α treatment to improve endometriosis-associated pain scores [130]. In a recent retrospective study, endometrioma patients receiving anti-TNF-α treatment etanercept with ART had improved clinical pregnancy rate and nonsignificantly increased liver birth rate [102]. Another study attempted to target IL-6 in a rat endometriosis model and demonstrated that tocilizumab treatment decreased the endometriotic lesion size and VEGF expression in the ectopic and eutopic endometria [131]. Instead of inhibiting proinflammatory cytokines, Quattrone et al. showed that targeted delivery of anti-inflammatory cytokine IL-4 could also reduce ectopic lesion development [132].

Another area where targeting cytokines could be of interest is prevention of postoperative adhesion formation in endometriosis. Pelvic anatomical distortion and adhesion create a physical disturbance between the ovary and fallopian tube, causing tubal blockage and interference with ovum pick-up and transport. Postoperative adhesion is reported in over 60% of patients undergoing gynecological surgery and may present symptoms such as bowel obstruction, abdominal pain, and infertility [133]. Endometriosis may increase the risk of postoperative adhesion [134]. Reduction of postoperative adhesion and inflammation is necessary because adhesion and COX-2 overexpression are significant risk factors for endometriosis recurrence after surgery [135]. 

Pelvic adhesion formation in endometriosis is largely attributed to local inflammatory reactions and an impaired fibrinolytic system [136]. Levels of proinflammatory cytokines IL-1, IL-6, TNF-α, TGF-β, and VEGF are correlated with adhesion presence and severity of endometriosis [137,138]. TGF-β is a major driver of fibrosis in endometriosis and can promote fibroblast to myofibroblast transdifferentiation via the Smad-dependent and Smad-independent signaling pathways [139]; it also promotes epithelial–mesenchymal transition (EMT) of endometrial cells and increases their migration ability [140,141]. Blockade of TGF-β and its affiliated targets reverses EMT and myofibroblast transdifferentiation in endometriosis and offers a potential therapeutic target [142,143]. Additionally, an in vivo study using an experimental endometriosis model showed that the presence of IL-1β in the peritoneal fluid of endometriosis patients could contribute to surgery-related adhesion, and inhibiting IL-1β with IL-1 receptor antagonist could significantly reduce postoperative adhesion [144].

### 3.3. Statins

Statins are a class of drugs that act as inhibitors of 3-hydroxy-3-methylglutaryl-coenzyme-A (HMG-CoA) reductase. They are commonly used in clinical practice to treat hypercholesterolemia but are increasingly recognized for their anti-inflammatory effects [145]. One study showed that simvastatin had a comparable effectiveness to GnRH agonists in reducing postoperative pain recurrence in endometriosis patients [106]. In preclinical studies, statins decreased immune cell migration and adhesion by reducing chemokine production and surface adhesion molecule expression [146]. Statins may also reduce T-cell activation by decreasing the expression of major histocompatibility complex molecules and altering lipid raft formation [146]. On a molecular level, statins can decrease NFκB nuclear translocation and AP1 activity, thereby reducing inflammation [146]. In vivo animal studies revealed that statins could reduce endometriotic implant size by inhibiting proliferation and promoting apoptosis, reducing VEGF and MMP-9 expression, and improving the pelvic adhesion extent [147]. Studies of its anti-inflammatory effect in endometriosis have shown that atorvastatin can reduce expression of MCP-1, COX-2, RAGE, EN-RAGE, and VEGF, as well as oxidative stress [147,148,149]. However, the effect of statins on fertility outcomes remains less certain because no clinical study has investigated it in an endometriosis context; nevertheless, preclinical studies have suggested that statins could enhance ovarian granulosa cell apoptosis and inhibit steroidogenesis [150,151]. Furthermore, because statins inhibit COX-2 activity in similar manner to aspirin, whether statins also negatively affect oocyte maturation and embryo quality should be verified [152]. 

### 3.4. Tyrosine Kinase Inhibitors

Both the MAPK and phosphoinositide 3-kinase (PI3K)/Akt pathways are enriched in the eutopic and ectopic endometria [153,154,155], and their relationships with chronic inflammation have been established [156]. These pathways are involved in inflammation, cell cycle progression, cell proliferation and migration, angiogenesis, and fibrosis [156]. Overactivation of these pathways compromises endometrial cells’ ability to decidualize, promote progesterone resistance, and increase proliferation and migration of endometrial cells [71]. The MAPK pathway is also involved in IL-1β- and TNF-α-mediated production of IL-6 and IL-8 [156]. Consequently, drugs that inhibit these pathways have potential for application in endometriosis treatment [157,158]. In this regard, tyrosine kinase inhibitors (TKIs) such as sorafenib, sunitinib, pazopanib, and vemurafenib have been tested [159]. These TKIs target Raf kinase and tyrosine kinase receptors such as vascular endothelial growth factor receptor (VEGFR) and platelet-derived growth factor receptor β (PDGFRβ) and have been used for their antiproliferative and antiangiogenesis effects to treat various tumors [160]. In addition, their immunomodulatory effects have been recognized [161]. Santulli et al. showed that inhibiting B-RAF and MAPK with vemurafenib decreased the endometriotic size in an animal model, with a concomitant decrease in COX-2 level and cell cycle progression [162]. Sunitinib was able to decrease the endometriotic implant volume and adhesion level in an experimental animal model, presumably by inhibiting VEGFR and enhancing apoptosis [159,163]. When various multitargeted TKIs were compared, each seemed to have a different effect on endometriosis extent, VEGF expression, and CD117 expression [164]. Pazopanib and sunitinib significantly reduced endometriotic lesions, whereas sorafenib had no significant effect [164]. Three drugs (pazopanib, sunitinib, and sorafenib) reduced VEGF expression but only pazopanib and sunitinib reduced CD117 expression [164]. Studies published to date have found no negative effects of these drugs on ovarian reserve, but further studies are necessary to assess the immune profile after drug administration and delineate the differential applicability of TKIs for enhanced infertility treatment [165]. 

### 3.5. Prostaglandin E2 (PGE2) Inhibitors

In endometriosis patients, there is a significant increase in PGE2 in peritoneal cavity and eutopic endometrium [166,167]. PGE2 is involved in pathogenesis of endometriosis and affects oocyte maturation, ovulation, and fertilization. PGE2 exerts its biological functions via four G protein-coupled receptors EP1, EP2, EP3, and EP4 [166]. Both EP2 and EP4 act through Gs-coupled effects, while EP1 acts via Gq-coupled pathway [166]. EP3 exerts its function via both Gs- and Gq-coupled pathways [166]. The secretion of PGE2 are regulated by passive diffusion, prostaglandin transporter protein (PGT), and multidrug resistant protein 4 (MRP4) [168]. Banu et al. demonstrated that EP2 and EP4 were abundantly expressed, while EP1 and EP3 were lowly expressed in ectopic and eutopic endometrial tissues [169]. Another study found that EP3, EP4, PGT, and MRP4 were overexpressed in ectopic endometrium [168]. Selective inhibition of EP2 and EP4 were demonstrated in preclinical studies to reduce progression of endometriotic lesion and modify the estrogen-dominant and progesterone-resistant microenvironment of eutopic endometrium with decrease in PGE2, E2 biosynthesis, and restoration of PR expression [170]. Inhibition of EP2 and EP4 also decreased inflammatory environment by reducing COX-2, IL-1β, IL-6, and TNF-α expression in both epithelial and stromal compartments of eutopic endometrium [170]. This study suggests the potential use of EP2/EP4 inhibitors to restore endometrial receptivity for improved implantation.

### 3.6. Antioxidants

Excessive oxidative stress negatively impacts reproductive processes, causing cellular and molecular damages and influencing oocyte maturation, fertilization, and implantation. Oral antioxidant supplements that can modulate inflammatory mediator expression, improve ovulation and endometrial receptivity have therefore attracted growing interests. A recent Cochrane systematic review evaluated 50 randomized controlled studies and concluded that coenzyme Q10 and combined antioxidants (containing multiple vitamins, minerals, and trace elements) may benefit outcomes of infertility treatments [171]. Although the quality of current data is limited, the finding suggested that oral antioxidant supplements can improve live birth and clinical pregnancy rates in subfertile women [171]. Unfortunately, this review did not assess studies targeting endometriosis population.

Women with endometriosis have overproduction of inflammatory mediators, higher oxidative stress induced by ROS and free radicals, and lower total antioxidant potential [172]. One randomized controlled trial showed that combined vitamin E and vitamin C supplementation reduced peritoneal inflammatory markers and improved painful symptoms in endometriosis patients with pelvic pain [113]. Addition of other antioxidative minerals like zinc, copper, and selenium to diet also correlates with positive effects such as improved antioxidant enzyme activity and diminished oxidative stress [173]. A phase II randomized controlled trial demonstrated that oral intake of melatonin was associated with improved sleep quality, reduced pelvic pain levels, and decreased brain-derived neurotrophic factor level in endometriosis women with chronic pelvic pain and dysmenorrhea [114]. Other natural antioxidants include curcumin and resveratrol [174,175]. While only few studies were done, the results showed significant reduction in serum ROS and lipid peroxidation and an increase in total antioxidant capacity [174,175]. Further clinical trials are required to accrue more evidence. Other nondietary antioxidant treatments like cerium oxide nanoparticles, nanoceria, have been studied in an endometriosis animal model [176]. Positive results included decreased systemic oxidative stress, reduced endometriotic lesion angiogenesis, and higher oocyte quality [176].

## 4. Cell-Based Therapy for Endometriosis Treatment

### 4.1. Stem Cells in Endometriosis Pathogenesis

The regenerative potential of endometrial tissue during each menstrual cycle has prompted research into stem cells present in the endometrium. Although Sampson’s retrograde menstruation theory is the best-known explanation of the pathogenesis of endometriosis, it does not explain the occurrence of extraperitoneal endometriotic lesions. Studies are increasingly suggesting an association between adult stem cells and endometriosis progression, and such evidence has recently been reviewed [177,178]. Endometrium-derived stem/progenitor cells may be shed through the fallopian tube, thereby establishing endometriotic implants [177]. Endometrial stem or progenitor cells have been identified as clonogenic cells in human endometria and as label-retaining or CD44^+^ cells in endometria of mice. Several markers for isolation of endometrial mesenchymal stem/stromal cells (emMSCs) have been used, including CD146, PDGFRβ, and SUSD2 [177]. These emMSCs have been isolated from eutopic and ectopic endometria in human biopsies [179,180,181,182,183,184]. Studies have shown that mesenchymal stem/stromal cells (MSCs) derived from endometriotic implants have higher expression levels of pluripotent octamer-binding transcription factor 4 (OCT4) and responded better to niche cytokines such as TGF-β to increase proliferation, migration, invasion, and angiogenic properties compared with MSCs derived from a eutopic endometrium or healthy control endometrial tissue [140,141,179,182]. Because ectopic emMSCs have greater proliferative, migratory, and angiogenic abilities than eutopic emMSCs with higher expression of HIF-1α and VEGF, preclinical studies have proposed using TKIs such as sorafenib to target ectopic emMSCs [185].

MSCs located within endometriotic ovarian cysts express higher levels of immunosuppressive proteins indoleamine 2,3-dioxygenase 1 (IDO-1), COX-2, and HO-1, and proinflammatory chemokine C-X-C motif chemokine ligand 12 (CXCL-12) compared with eutopic endometria [186]. Moreover, MSCs derived from these endometriotic cysts control monocyte differentiation into immunosuppressive M2 macrophages [186]. Therefore, ectopic endometrial MSCs may contribute to an immunosuppressed environment in the pelvic cavity, thereby enabling immune escape of ectopic lesions and supporting their growth in endometriosis [186].

Adult stem cells of extra-uterine origin contribute to endometriosis pathogenesis. Bone marrow-derived cells (BMDCs) are a collection of hematopoietic stem cells, MSCs, and endothelial progenitor cells; these cells play a crucial role in tissue repair because they can be recruited to distant inflamed sites and either transdifferentiate to replenish injured cell types or modulate the healing process through paracrine effects [187]. In addition, BMDCs participate in normal endometrial regeneration in the menstrual cycle and are incorporated into epithelial, stromal, and endothelial compartments of the endometrium [188]. Whether BMDCs can transdifferentiate to become functional components such endometrial gland is still under debate [189,190].

BMDCs can be attracted to an endometriotic lesion via the signaling axis of CXCL-12 and its receptor, C-X-C motif chemokine receptor 4 (CXCR4), and estradiol enhances chemoattraction by increasing both the endometrial stromal cell secretion of CXCL-12 and the BMDC expression of CXCR-4 [191]. Endometriotic lesions also compete with the eutopic endometrium for BMDCs, and reduced recruitment of BMDCs in the eutopic endometrium possibly impairs its regenerative capability, leading to infertility [192]. Furthermore, estrogen deprivation therapy with GnRH agonist and aromatase inhibitor restores recruitment of BMDCs in the eutopic endometrium, with greater regression of ectopic lesions, whereas progestin does not significantly affect BMDC recruitment, suggesting that targeting stem cells could be a potential treatment strategy [193].

Anglesio et al. demonstrated that 26% of deep-infiltrating endometriotic lesions harbored cancer-associated somatic mutations that were confined within the epithelial compartment and not found in the stromal compartment [194]. Although further research is required for validation, one plausible explanation for this is that ectopic lesions may be derived from two cellular origins, namely initial establishment of the epithelial compartment by endometrial progenitor cells harboring cancer driver mutation, followed by stromal compartment establishment by BMDCs [194].

### 4.2. Inflammatory Niche–Induced Stemness Re-Expression in Endometriosis Pathogenesis

Evidence suggests that microenvironmental inflammatory cytokines and growth factors may cause significant changes in cellular behavior, promoting stemness, EMT, invasion, and malignant transformation. The interplay between inflammatory niche and stemness has been widely studied in cancer biology. Factors such as IL-1β, IL-6, IL-17, and TNF-α have been implicated in the maintenance of cancer stemness and promotion of invasive phenotypes in breast cancer [195], ovarian cancer [196], colon cancer [197], hepatocellular carcinoma [198,199], and thyroid cancer [200]. These inflammatory cytokines can be secreted by tumor-associated immune cells, cancer stromal cells, or cancer cells and function via NFκB- or STAT3-mediated pathways [201,202]. Inflammation may also promote stemness properties by stimulation of growth factor production. For example, IL-6 can stimulate IGF-1 production and enhance stemness of HBV-associated hepatocellular carcinoma cells, leading to early recurrence [198]. 

MSCs themselves are regulated by the inflammatory niche. The cytokine secretion profiles of MSCs change with stimulation by inflammatory signals such as lipopolysaccharides (LPS), and MSCs of different origins respond differently to the same stimulus [203]. For example, BM-MSCs secrete higher levels of VEGF-A, CXCL-12, IL-1RA, IL-6, MCP-1, and MIP-1α after LPS stimulation compared with emMSCs [203]. Proinflammatory cytokines such as IL-6 can maintain BM-MSCs in their proliferative and stemness state by activating the ERK1/2 pathway [204]. In addition, transient treatment of dental progenitor cells with TNF-α increased their stemness phenotype [205]. In an inflammatory microenvironment, MSCs may exhibit a profibrotic phenotype through higher expression of MMPs, IL-1, IL-6, TNF-α, and type-1 collagen [206]. The effects of cytokines on MSCs are not always unidirectional because a high TNF-α concentration may be detrimental to MSC survival [207]. TGF-β secreted by stromal fibroblasts appears to promote differentiation of emMSCs into endometrial stromal fibroblasts [208]. Inhibiting TGF-βR enhances emMSC surface marker expression, promotes cell proliferation, and prevents apoptosis of emMSCs [208]. The inflammatory niche may also contribute to endometriosis, which is characterized by increased stabilization of HIF-1α and HIF-1α-induced expression of VEGF and MMP-9 in eutopic and ectopic endometria [209,210,211,212]. 

### 4.3. Immunomodulation of MSCs

MSCs are a mixture of various stromal cell types that have pleiotropic properties and are believed to participate in the tissue- and wound-healing process [213]. Because of their self-renewal and differentiation potential, they have been regarded as a potential cell type for regenerative medicine. Moreover, they have been recognized as negative for hematopoietic cell markers with expression of CD90, CD105, CD44, CD73, CD9, and CD80 [214]. Sources of MSCs are diverse; they have been isolated from numerous sources such as bone marrow (BM), adipose tissue, tonsils, umbilical cord, skin, dental pulp, placenta, and endometrium. Pericytes—perivascular cells found in multiple organs and expressing PDGF-Rβ, CD146, NG2, and typical MSC markers—have been suggested as possible progenitors of non-BM-MSCs [215].

MSCs have different effects on different immune cell types, including both innate and adaptive immune systems. They secrete factors such as IDO, PGE2, NO, TGF-β, and HGF to reduce activation and cytotoxicity of NK and T-cells [216]. Moreover, these factors decrease the proliferation and activation of B- and T-cells, leading to reduced antibody and cytokine production. MSCs increase regulatory T-cell numbers, inhibit dendritic cell maturation and the macrophage M1 phenotype, promote polarization of the M2 phenotype, and suppress mast cell degranulation [217]. These anti-inflammatory changes have prompted researchers to investigate MSCs for treatment of inflammatory diseases.

Although numerous studies have focused on the immunosuppressive effects of MSCs and their potential application in treating inflammatory diseases, they also have immunostimulatory properties. MSCs are sensitive to their microenvironment and their interaction with it determines their role in enhancing or suppressing inflammation. For example, the immunosuppressive effect of MSCs on T-cells depends on IFN-γ, IL-1, and TNF-α induction of NO production; however, low concentrations of these cytokines cannot induce sufficient NO production to suppress T-cell proliferation, leading to the activation of T-cell responses [218]. IL-6 expression by MSCs affects their ability to polarize macrophages on cell contact [219]. IL-6 in combination with PGE2 and IDO drives M1 polarization [219]. In the absence of IL-6, differentiation toward the M2 phenotype because of TNF-α and IFN-γ exposure is favored [219]. Further studies are required to clarify how MSCs might be consistently applied as immunomodulatory agents in clinical use.

Different tissue origins of MSCs are available for research and clinical use. BM represent a common MSC source in clinical practice, with early success in treatment of graft-versus-host disease [217]. However, extraction of BMMSCs requires an invasive and painful BM aspiration procedure, and the number of BMMSCs declines with donor age. Adipose tissue-derived (AT) MSCs have gained significant interest as liposuction has become an increasingly common operation, and the greater stem cell yield from adipose tissue allows faster expansion of ATMSCs for therapeutic use [220]. Neonatal stem cells from biological materials obtained after birth, namely placenta, umbilical cord, and umbilical cord blood, are also attractive cell therapy options for autologous and allogeneic transplantation. Although MSCs of different origins have similar morphology, they can have different biological properties, including epigenetic regulation, gene expression profile, surface receptor expression, proliferation and differentiation capacity [221,222,223]. However, different studies have reported inconsistent comparison results of different MSC origins, possibly due to differences in cell culturing conditions.

MSCs derived from different sources seemed to have different immunomodulatory effects on different immune cell types. When comparing BMMSCs with ATMSCs, only BMMSCs suppressed NK cell cytotoxic activity, but ATMSCs were more potent at inhibiting dendritic cell differentiation, mononuclear cell proliferation, and B cell immunoglobulin secretion [224,225,226,227]. The more potent immunomodulatory capacity of ATMSCs could be attributed to higher level of IDO expression in response to IFN-γ and higher levels of immunosuppressive cytokine IL-10 secretion when co-cultured with monocytes [226]. While both BMMSCs and ATMSCs inhibited B cell activity, umbilical cord MSC seemed to have little effect [228]. Another study showed that, when compared with BMMSC and ATMSC, umbilical cord Wharton’s jelly MSCs expressed the lowest level of immune response markers MHC II, JAG1, TLR4, TLR3, NOTCH2, and NOTCH3 and had the most inhibitory effect on T cell proliferation [223]. Although placenta-derived MSCs also have immunomodulatory effects, their relative immunomodulatory capacity compared with other tissue origins is contradictory [229,230]. MSCs isolated from neonatal sources like placenta, umbilical cord, and umbilical cord blood also express high level of HLA-G, a critical surface antigen responsible for inducing immunotolerance and allowing fetal tissues to coexist with maternal immune system during pregnancy [231,232]. The presence of HLA-G on neonatal MSCs may therefore make them favorable cell therapy candidates for allogeneic transplantation.

### 4.4. Safety and Efficacy of Stem Cell Therapy in Endometriosis-Associated Infertility

Development of MSC-based delivery vectors has generated considerable interest because of their tropism toward inflamed lesion foci such as tumors. Stem cell therapy has been applied to treat infertility, especially Asherman’s syndrome [233], which is characterized by endometrium adhesion and fibrosis due to tuberculosis infection or following hysteroscopic procedures such as dilation and curettage; such patients present with absent menstruation and infertility. The objective of MSC transplantation therapy is to reduce the degree of inflammation after adhesiolysis and promote endometrial regeneration [234]. Autologous in utero BM stem cell transplantation facilitated IVF in a case report of a woman with severe endometrial adhesion following dilation and curettage [235]. In addition, CD133+ bone marrow-derived cell transplantation enabled endometrial thickness growth, increased neovascularization, and menses restoration in a cohort study of 11 Asherman’s syndrome patients [236]. MSC transplantation also improved ovarian function in premature ovarian insufficiency and aging animal models by increasing folliculogenesis, decreasing granulosa cell apoptosis and the extent of ovarian fibrosis, and modulating cytokine expression [237,238,239].

Safety always takes priority over efficacy. Application of MSCs for treating endometriosis, and in particular endometriosis-associated infertility, has caused controversy because of stem cell involvement in the disease’s pathogenesis [240]. For example, several reports have indicated that bone marrow-derived MSCs can differentiate into carcinoma-associated fibroblasts and promote tumor growth, possibly involving niche TGF-β, CXCL-12, or IL-6 [241,242,243,244,245,246]. Additionally, MSCs from adipose tissue have been demonstrated to promote cancer proliferation and metastasis through STAT3 activation [247,248]. The effects of different MSC sources on gynecological and breast cancer are summarized in Table 2. Because endometriosis behaves similarly to cancer in many ways and correlates with gynecologic cancer occurrence, considering the risk of disease promotion is vital when determining the optimal cell therapy type. Moreover, carefully designing a cell handling and delivery protocol is essential because different ex vivo expansion methods could affect MSC phenotypes in different manners [249], and systemic infusion provides a better uterine engraft than does local injection [250].

Because MSCs can be derived from multiple organs, the choice of which one to use for clinical application is a challenging problem that requires assessment of safety and efficacy. MSCs of different origin can have different responses to the same niche. Khatun et al. demonstrated that although BM-MSCs and emMSCs exhibit high proliferative and migratory capabilities, BM-MSCs secrete higher levels of cytokines under LPS stimulation, including IL-6, CXCL-12, MCP-1, RANTES, and VEGF-A [203]. Therefore, emMSCs appear to have the greatest potential for use in endometriosis treatment and have been evaluated for their potential use as a cell therapy targeting vector for endometriosis treatment [262]. They did not generate tumors in subcutaneous tumorigenicity in vivo, and were shown to accumulate in an endometriosis implant after systemic intravenous injection [262]. However, no significant differences in endometriotic lesion size, VEGF expression, or microvascular density at sacrifice were observed. Koippallil Gopalakrishnan et al. studied the use of emMSCs as a cell-based targeting vector for endometriosis. These endometrial MSCs were engineered to express sFlt-1—the soluble truncated form of VEGFR—to inhibit angiogenesis. The engineered emMSCs gathered in the endometriotic implant after systemic injection and reduced the implant size and microvessel density [263]. Although few studies have investigated endometrial MSCs, results suggest that they are a safe cell type that does not promote endometriosis progression [262,263].

Other cell origins such as BM and umbilical cord blood have been investigated. BMDCs were the first to be tried in an endometriosis model [264]. Although BMDCs decreased in situ TNF-α and VEGFR levels, they did not affect lesion size [264]. In another study, bone marrow-derived MSCs were injected locally into an endometriotic implant in a rabbit endometriosis model [265]. The MSC group had significantly more intra-abdominal adhesions and a lower pregnancy rate than did the control group, and no reduction in implant size with MSC treatment was noted [265]. When human umbilical cord mesenchymal stem cells were used to treat cultured endometriotic cells, the cytokine expression of IL-1β and proliferation rate of endometriotic cells increased in vitro [266].

## 5. Conclusions

**Figure 2:**
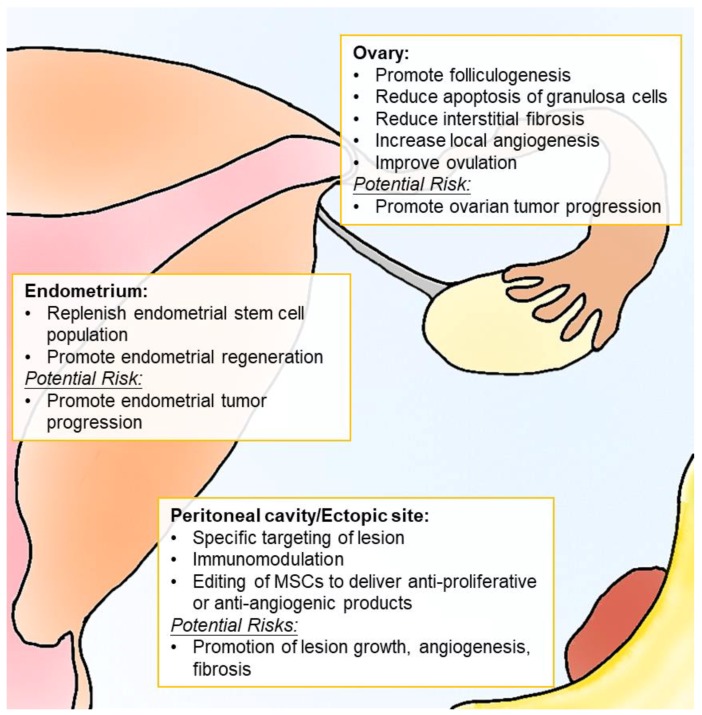
Mesenchymal stem/stromal cell (MSC)-based therapy for treatment of endometriosis-associated infertility: safety and efficacy.

Medical therapies for endometriosis commonly focus on relieving its painful symptoms as opposed to curing the disease. Between 5% and 59% of patients undergo ineffective treatment, and 17–34% experience recurring symptoms after treatment cessation [267]. Developing novel therapies would benefit patients with poor response by improving quality of life and infertility outcomes. Therefore, many medical therapy strategies have been devised to target the dysregulated immune system that characterizes endometriosis, but none has proven sufficiently effective to be incorporated into a standard management protocol. MSC-based cell therapy offers an attractive option for addressing the infertility problem of endometriosis patients because of its tropic and immunomodulatory effects. MSC-based cell therapies have been tested in infertility treatment for patients with Asherman’s syndrome and premature ovarian failure. However, much remains to be explored where patient safety is concerned. Careful selection and handling of the MSC source to target endometriotic lesions without promoting lesion growth is crucial. Different sources of MSC may respond differently to the inflammatory niche, whereas the same MSCs may alter their behaviors because of their plasticity. Therefore, defining different cytokine–receptor interactions for different MSC origins is necessary. Gene-editing of MSCs to express antiproliferative or antiangiogenic products may also be explored to take full advantage of MSC-based cell therapy (Figure 2).

## Figures and Tables

**Table 1:** Potential immunomodulatory pharmaceutical agents for endometriosis treatment in clinical studies.

Treatment	Proposed Mechanism	Indication	Outcome	Comments
**Anti-TNF-α Treatment**				
Etanercept	Binds and inhibits TNF-α	Infertility	Significantly higher clinical pregnancy rate in patients receiving etanercept	Ref. [102]
Infliximab	Binds and inhibits TNF-α	Pain	No significant effect on pain or lesion size	Phase II trial Ref. [103]
**Cytokine Treatment**				
Recombinant interleukin-2 (rIL-2)	Enhances cytotoxic activity of macrophages and NK cells	Endometrioma postdrainage recurrence prevention	Significantly longer time to disease recurrence with favorable symptom improvement in rIL-2 and GnRH agonist combination group	Ref. [104]
Interferon-α-2b	Enhances cytotoxic activity of macrophages and NK cells	Postoperative recurrence prevention	No improvement in disease recurrence	Ref. [105]
**Angiogenesis Inhibitor**				
Simvastatin	Inhibits proliferation and angiogenesis	Postoperative pain	No significant difference from GnRH agonist group	Ref. [106]
Quinagolide	Dopamine receptor agonist for treatment of hyperprolactemia; also has VEGFR2 inhibition effect	Hyperprolactinemic patients with endometriosis	Decreased lesion size with downregulation of angiogenesis markers	Ref. [107]
Cabergoline	Dopamine receptor agonist for treatment of hyperprolactemia; also has VEGFR2 inhibition effect	Pain	N/A	Phase II trial; Recruiting; Clinical Trials.gov: NCT00115661
**Immunomodulatory and Anti-Inflammatory Agents**				
Pentoxifylline	Nonselective phosphodiesterase inhibitor; reduces platelet aggregation through platelet phosphodiesterase inhibition; inhibits TNF-α and leukotriene synthesis	Infertility in mild/moderate endometriosis	Nonsignificant increase in cumulative probability of pregnancy in patients receiving pentoxifylline	Phase III trial; Clinical Trials.gov: NCT00632697; Ref. [108]
		Infertility	No significant difference in pregnancy rate or disease recurrence	Ref. [109]
		Postoperative pain	Improved pain score at 2–3 months after surgery	Ref. [110]
Pioglitazone	PPAR-γ agonist; inhibits inflammatory cytokine production and NFκB activity	Infertility	Nonsignificant increase in clinical pregnancy rate; significant increase in embryo implantation rate	Ref. [111]
Rosiglitazone	PPAR-γ agonist; inhibits inflammatory cytokine production and NFκB activity	Pain	Terminated/withdrawn due to adverse cardiovascular events	Clinical Trials.gov: NCT00115661/NCT00121953
Metformin	Suppresses inflammatory response and aromatase activity; decreases local estrogen production	Pain and infertility	Improved pregnancy rate; improved symptom score	Ref. [112]
Resveratrol	Inhibits hypoxia-mediated ERK1/2, AKT, and MMP2/9 activity	Pain	Nonsignificant decrease in pain score and serum CA-125 level	Phase IV trial; Clinical Trials.gov: NCT02475564
EGCg	Inhibits ROS-induced NFκB activation and MAPK, JNK, and p38 signaling; inhibits angiogenesis	Pain	N/A	Phase II trial; Recruiting; Clinical Trials.gov: NCT02832271
**Antioxidants**				
Vitamins E and C	Antioxidative activity; decrease peritoneal inflammation	Pain	Improved pain score; decreased peritoneal RANTES, IL-6, and MCP-1	Ref. [113]
Melatonin	Antioxidative activity	Pain	Improved pain score; decreased analgesic use; improved sleep quality	Phase II trial; Ref. [114]

EGCg, Epigallocatechin gallate; GnRH, gonadotropin-releasing hormone; IL-6, interleukin-6; MAPK, mitogen-activated protein kinase; MCP-1, monocyte chemoattractant protein-1; MMP, matrix metalloproteinase; NK, natural killer; PPAR-γ, peroxisome proliferator-activated receptor gamma; RANTES (CCL5), regulated on activation, normal T cell expressed and secreted; ROS, reactive oxygen species; TNF-α, tumor necrosis factor alpha; VEGFR2, vascular endothelial growth factor receptor 2.

**Table 2:** Promotive and suppressive effects of MSCs in gynecological and breast cancers.

Cancer type/MSC Source	Surface marker	Effect	Factors/Mechanisms	Ref.
**Ovarian cancer**				
Omental adipose tissue of healthy donor	CD73^+^ CD90^+^ CD105^+^ CD34^−^	Increased tumor growth and metastasis	ATMSC increased tumor cell secretion of MMP2 and MMP9	[251]
Umbilical cord Wharton’s jelly of healthy donor	CD44^+^ CD90^+^ CD105^+^ CD34^−^ HLA^−^	Decreased tumor growth	WJSC increased tumor cell apoptosis	[252]
Menstrual blood of healthy donor	CD73^+^ CD90^+^ CD34^−^	Decreased tumor growth and angiogenesis	emMSCs induced tumor cell cycle arrest, increased tumor cell apoptosis, decreased AKT phosphorylation, and promoted FoxO3a nuclear translocation of tumor cells	[253]
**Endometrial Cancer**				
Bone marrow of healthy donor	CD29^+^ CD44^+^ CD73^+^ CD90^+^ CD105^+^ EpCAM^−^ CD11b^−^ CD34^−^ CD45^−^	Increased tumor growth	BMMSC-secreted high level of VEGF, FGF, and SDF1-α; Tumor-secreted IL-8 and CXCL-1 attracted BMMSCs to the tumor site	[254]
Omental adipose tissue of healthy donor	CD73^+^ CD90^+^ CD105^+^ CD34^−^	Increased tumor growth and metastasis	ATMSC-secreted IL-6 activated STAT3 pathway in tumor cells	[247]
Omental adipose tissue of gynecologic cancer patients, subcutaneous adipose tissue of healthy donor	CD29^+^ CD44^+^ CD73^+^ CD90^+^ CD105^+^ EpCAM^−^ CD11b^−^ CD34^−^ CD45^−^	Increased tumor growth (omental ATMSCs); No significant tumor promotion with subcutaneous ATMSCs	Omental ATMSCs secreted high level of VEGF, FGF, and SDF1-α; Tumor-secreted, IL-8 and CXCL-1 attracted ATMSCs to the tumor site	[254]
**Cervical Cancer**				
Amniotic fluid in second trimester of gestation	CD73^+^ CD90^+^ CD105^+^ CD14^−^ CD34^−^ CD45^−^ HLA^−^	Increased tumor growth	Genetically modified IFN-α-expressing AFMSCs suppressed tumor growth	[255]
**Breast Cancer**				
Bone marrow of healthy donor	CD105^+^ CD31^+^ CD34^−^ CD133^−^	Increased tumor growth and metastasis	Tumor increased BMMSC–secreted CCL-5 (RANTES) to increase cell motility, invasion, and metastasis	[256]
Bone marrow of healthy donor	Not mentioned	Varying effect in tumor growth and metastasis for different breast cancer cell lines	BMMSC-secreted IL-17 and tumor-expressed IL-17R may stimulate migration of metastatic cancer cells; Tumor-secreted TGF-β1 attracted BM-MSCs	[257]
Subcutaneous abdominal adipose tissue of healthy donor	CD29^+^ CD73^+^ CD90^+^ CD105^+^ CD166^+^ CD11b^−^ CD31^−^ CD34^−^ CD45^−^ HLA-DR^−^	Varying effect of tumor growth and angiogenesis for different breast cancer cell lines	ATMSC-secreted CXCL-1 and CXCL-8 promoted tumor angiogenesis	[258]
Umbilical cord of healthy donor	CD13^+^ CD29^+^ CD44^+^ CD73^+^ CD90^+^ CD105^+^ CD106^+^ CD166^+^ ABC^+^ HLA^−^ CD14^−^ CD31^−^ CD34^−^ CD38^−^ CD45^−^ HLA-DR^−^	Increased migration and metastasis (MCF-7)	UCMSC-secreted IL-6 and IL-8 promoted tumor cells to secret IL-6 and IL-8 to increase migration and mammosphere formation	[259]
Umbilical cord of healthy donor	CD29^+^ CD44^+^ CD54^+^ CD73^+^ CD90^+^ CD105^+^ CD11b^−^ CD19^−^ CD31^−^ CD34^−^ CD45^−^ HLA-DR^−^	Decreased tumor growth and angiogenesis (MDA-MB-231)	Increased apoptosis	[260]
Umbilical cord Wharton’s jelly of healthy donor	CD44^+^ CD90^+^ CD105^+^ CD34^−^ HLA^−^	Decreased tumor growth and migration	Increased apoptosis	[252]
Amniotic tissue of healthy donor	Not mentioned	Decreased tumor growth	AMESCs secreted TNF-α, TNF-β, TGF-β, IFN-γ, IL-2, IL-3, IL-4, M-CSF, and IL-8	[261]

AFMSC, amniotic fluid-derived mesenchymal stem cell; AMESC, amniotic membrane-derived epithelial stem cell; ATMSC, adipose tissue-derived mesenchymal stem cell; BMMSC, bone marrow-derived mesenchymal stem cell; CD, cluster of differentiation; emMSC, endometrial mesenchymal stem cell; FGF, fibroblast growth factor; IL, interleukin; M-CSF, macrophage colony-stimulating factor; MMP, matrix metalloproteinase; MSC, mesenchymal stem cell; TGF-β, transforming growth factor beta; TNF, tumor necrosis factor; UCMSC, umbilical cord-derived mesenchymal stem cell; VEGF, vascular endothelial growth factor; WJSC, Wharton’s jelly stem cell.
